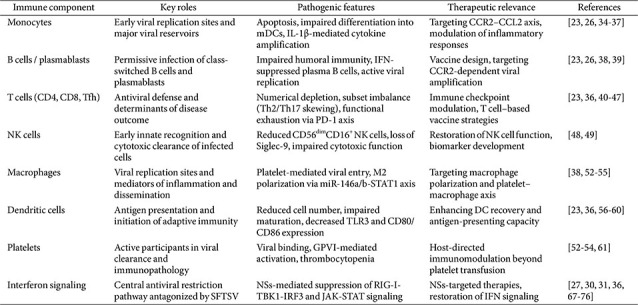# Immune Cells at the Frontline of SFTSV Infection

**DOI:** 10.4014/jmb.2511.11002

**Published:** 2026-02-24

**Authors:** Sungjun Park, Chonsaeng Kim

**Affiliations:** Center for Infectious Disease Vaccine and Diagnosis Innovation (CEVI), Korea Research Institute of Chemical Technology (KRICT), Daejeon, Republic of Korea

**Keywords:** Severe fever with thrombocytopenia syndrome virus (SFTSV), Tropism for immune cells, Monocytes, B cells, T cells

## Abstract

Severe fever with thrombocytopenia syndrome virus (SFTSV) is an emerging tick-borne *Dabie bandavirus* that causes hemorrhagic fever with high case fatality. Although numerous studies have examined specific aspects of SFTSV infection, an integrated framework that links viral immune cell tropism to immunopathogenesis remains lacking. The virus exhibits broad immune tropism, particularly infecting monocytes and B cells, which serve as major viral reservoirs and sources of inflammatory cytokines, while B cells additionally show impaired antibody production. T cells undergo numerical depletion and functional exhaustion, and dendritic cells lose antigen-presenting capacity in severe cases. Natural killer cells and macrophages also exhibit altered activation and polarization, contributing to both antiviral defense and immunopathology. A key viral protein, NSs, antagonizes host interferon signaling by sequestering TBK1, IRF3, and STAT1/2 into viral inclusion bodies, thereby suppressing type I and II IFN responses. Murine models with disrupted IFNAR signaling have been widely used to study SFTSV pathogenesis *in vivo*. In this review, we aim to integrate clinical, experimental, and molecular evidence to synthesize the roles and functional features of immune cells underlying SFTSV pathogenesis and highlight host-directed immunotherapeutic advances.

## Introduction

*Severe fever with thrombocytopenia syndrome virus* (SFTSV), an emerging tick-borne *Dabie bandavirus*, was reclassified by the International Committee on Taxonomy of Viruses (ICTV) as a member of the order *Bunyavirales*, family *Phenuiviridae*, and genus *Bandavirus* [1, 2]. First identified in China in 2009, SFTSV has since been reported in several East Asian countries including South Korea, Japan, and Vietnam [3-5]. SFTSV is transmitted primarily through the bite of *Haemaphysalis longicornis* ticks, but cases of human-to-human transmission via blood or bodily fluids have also been documented, particularly in healthcare settings [6-8].

Beyond human transmission, understanding the ecological distribution and maintenance of the tick vector is essential to assess the risk of viral spread. *H. longicornis*, the primary vector of SFTSV, is widespread across East Asia and has recently been established in multiple eastern U.S. states, raising concerns about its potential role in SFTSV emergence outside its traditional endemic regions [9, 10]. Surveillance studies have detected low but consistent SFTSV RNA positivity across all developmental stages of *H. longicornis*—including unengorged larvae—suggesting vertical (transovarial) transmission supports vector maintenance in nature [6, 11]. Moreover, a diverse range of domestic and wild animals have been shown to carry either viral RNA or seropositivity, indicating their roles as potential amplifying or reservoir hosts in the zoonotic cycle of SFTSV [12]. A deeper understanding of SFTSV's infection across hosts requires insight into its genomic structure and coding capacity.

**Fig. 1:**
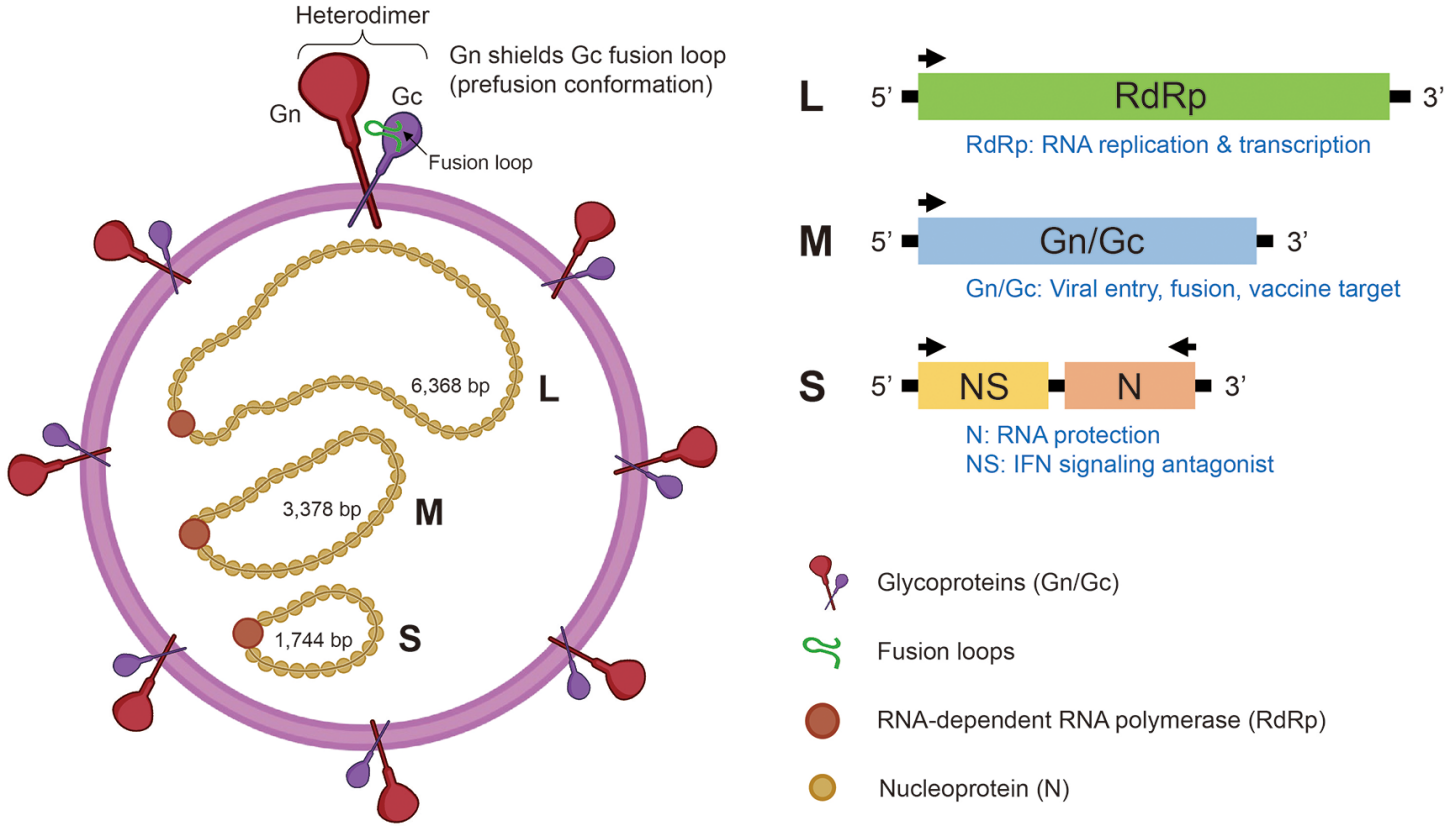
Genomic structure and encoded proteins of the SFTSV. SFTSV contains three genomic RNA segments: L, M, and S. The L segment encodes the RNA-dependent RNA polymerase (RdRp), essential for replication and transcription. The M segment encodes a glycoprotein precursor cleaved into Gn and Gc, which form heterodimers on the virion surface. Within this architecture, Gn shields the Gc fusion loop, stabilizing the prefusion conformation that serves as a major target for neutralizing antibodies. The S segment, in ambisense orientation, encodes the nucleocapsid protein (N), which supports RNA encapsidation, and the nonstructural protein NSs, which antagonizes interferon responses. The schematic shows the virion and protein-coding features of each segment [14-18].

Structurally, SFTSV possesses a tripartite, negative-sense RNA genome, characteristic of viruses within the order *Bunyavirales*. The genome consists of three segments—L, M, and S. The L segment (~6.3 kb) encodes the RNA-dependent RNA polymerase (RdRp), which mediates both viral genome replication and mRNA transcription [13]. The M segment (~3.4 kb) encodes a glycoprotein precursor that is cleaved by host proteases into two envelope glycoproteins, Gn and Gc [14]. These glycoproteins facilitate viral entry by binding to cellular receptors and mediating pH-dependent membrane fusion during endocytosis [15]. Recent single-particle cryo-electron microscopy (cryo-EM) and sub-particle reconstruction analyses have further elucidated their organization, revealing that Gn and Gc assemble into heterodimers, which in turn arrange into pentameric and hexameric peplomers forming an icosahedral lattice on the virion surface. Within this architecture, Gn shields the Gc fusion loops through intra- and inter-heterodimer interactions, thereby stabilizing Gc in its prefusion conformation [16]. In addition to their essential role in viral infectivity, Gn and Gc are highly immunogenic and represent promising targets for the development of neutralizing antibody-based vaccines [17]. Notably, the S segment encodes both the nucleocapsid protein (N) and the nonstructural protein NSs in an ambisense orientation. While N is essential for RNA encapsidation and replication complex formation, NSs functions as a potent interferon antagonist, thereby facilitating immune evasion [18]. The genomic structure of SFTSV is illustrated in Fig. 1.

Clinically, SFTSV infection leads to severe fever with thrombocytopenia syndrome (SFTS), which is characterized by high fever, thrombocytopenia, leukopenia, gastrointestinal symptoms, and in severe cases, multi-organ failure and hemorrhagic manifestations [19-22]. SFTSV infection has been associated with a highly variable case fatality rate, influenced by factors such as age, comorbidities, and geographic region, with some reports documenting rates exceeding 30% [1].

Fatal infection with SFTSV is characterized by profound immunological dysfunction affecting both innate and adaptive immune compartments. Song *et al*. (2018) demonstrated that extensive apoptosis of circulating monocytes and impaired differentiation of myeloid dendritic cells (mDCs) contribute to innate immune collapse. Simultaneously, infection and dysfunction of class-switched B cells and plasmablasts hinder effective humoral responses [23]. Together, these defects result in compromised antiviral immunity that strongly correlates with lethal disease outcomes. In addition to impaired antiviral responses, fatal cases of SFTSV infection are often marked by excessive and uncontrolled production of proinflammatory cytokines—including IL-6, TNF-α, and IFN-γ—leading to a cytokine storm that drives systemic inflammation, vascular leakage, and multi-organ dysfunction, and is positively associated with disease severity [24, 25].

Despite the growing body of literature on SFTSV infection, existing studies have largely focused on discrete aspects of disease biology, leaving the links between viral immune cell tropism, immune evasion, and immunopathogenesis insufficiently integrated. Here, we integrate evidence from human and experimental studies to construct an integrative framework that connects immune cell-specific roles with SFTSV pathogenesis, immune evasion, and therapeutic relevance.

The CCR2–CCL2 chemokine axis plays a central role in recruiting virus-permissive inflammatory monocytes to infected tissues, thereby promoting both viral dissemination and immunopathology [26]. Concurrently, the nonstructural protein NSs of SFTSV functions as a potent antagonist of host antiviral signaling. NSs actively sequesters key signaling intermediates—including RIG-I, TBK1, and IRF3—into virus-induced cytoplasmic inclusion bodies, thereby disrupting TBK1-mediated phosphorylation, IRF3 dimerization, and nuclear translocation. Consequently, type I interferon induction and downstream antiviral responses are effectively suppressed [27, 28]. A recent genome-wide CRISPR-Cas9 screen expanded our understanding of SFTSV entry. It reaffirmed CCR2 as a host factor and identified low-density lipoprotein receptor-related protein 1 (LRP1) as a novel viral receptor. LRP1 knockdown or inhibition significantly reduced SFTSV infection *in vitro* and *in vivo*. The viral glycoprotein Gn binds to the CL I and CL II domains of LRP1, and neutralizing antibodies against LRP1 decreased viral loads, limited tissue damage, and improved survival in a lethal mouse model—highlighting its potential as a therapeutic target [29]. Experimental mouse models—particularly those with disrupted interferon signaling, such as IFNAR1-knockout or IFNAR1–antibody-blocked mice—have been instrumental in elucidating the pathogenesis and immune evasion strategies of SFTSV [30, 31].

Given the increasing geographic spread of SFTSV, the lack of licensed vaccines or specific antiviral treatments, and the virus’s capacity for immune modulation, there is an urgent need to better understand the immunopathological mechanisms underlying SFTSV infection [32, 33].

## Monocytes as Viral Reservoirs

SFTSV preferentially targets hematopoietic and innate immune cells, with monocytes being among the earliest and most heavily infected populations. Monocytes act as both early replication sites and critical drivers of downstream immunopathology [34]. In fatal human cases, Song *et al*. (2018) demonstrated that widespread apoptosis of monocytes and their impaired differentiation into myeloid dendritic cells (mDCs) led to a profound collapse of the innate immune compartment. This collapse compromised antigen presentation and hindered the initiation of effective antiviral responses [23].

Consistent with these findings, monocytes have been identified as the predominant infected population among peripheral blood mononuclear cells (PBMCs). SFTSV-infected monocytes produce high levels of IL-1β, implicating them in the systemic inflammation and cytokine dysregulation that characterize severe disease [35]. Corroborating this observation, single-cell RNA sequencing of patient samples confirmed that monocytes and plasmablasts are the primary cell types harboring circulating SFTSV RNA [36].

Further insights into viral entry mechanisms have implicated the chemokine receptor CCR2, which is highly expressed on inflammatory monocytes and plays a key role in their mobilization and trafficking. CCR2 binds its cognate ligand CCL2 (also known as MCP-1), facilitating monocyte egress from the bone marrow and their directed migration toward inflamed or infected tissues along chemokine gradients [37]. A genome-wide CRISPR-Cas9 screening study identified CCR2 as an essential host factor for SFTSV entry, demonstrating that its N-terminal domain directly interacts with the viral particle. Moreover, CCR2 expression levels in peripheral blood are positively correlated with disease severity, indicating a dual role in promoting viral dissemination and amplifying inflammation *in vivo* [26].

Together, these findings establish monocytes as central orchestrators of SFTSV pathogenesis—contributing to both viral propagation and the excessive inflammatory milieu that underlies severe disease.

## B Cell Responses and Susceptibility to SFTSV Infection

Accumulating pathological, serological, and transcriptomic evidence indicates that B cells—particularly class-switched, plasmablast-lineage lymphocytes—play a pivotal role in the pathogenesis of SFTSV infection. Suzuki *et al*. (2020) showed through postmortem analyses that IgG^+^ B cells differentiating into plasmablasts in secondary lymphoid organs (*e.g.*, spleen and lymph nodes) were the predominant infected population in fatal cases [38]. These infected B cells were also detected in peripheral organs such as the lung, heart, and thyroid, suggesting systemic viral dissemination. Supporting these observations, a human plasmablastic lymphoma cell line (PBL-1), sharing a similar immunophenotype with these differentiated B cells, was shown to support SFTSV replication *in vitro*, providing a relevant infection model.

Complementing these histopathological findings, Song *et al*. (2018) reported that fatal SFTS cases were characterized by defective humoral immunity, notably the absence of virus-specific IgG and a significant reduction in functional plasmablasts. In contrast, survivors showed progressive expansion of class-switched IgM^-^IgG^+^ plasmablasts (CD19^+^IgD^-^CD27^high^) during the first two weeks of infection, whereas fatal cases exhibited a striking decline, even below levels in healthy controls—highlighting a failure to mount protective antibody responses [23].

In addition to postmortem and serological findings, transcriptomic approaches have further elucidated the role of B cells in SFTSV infection. Using single-cell transcriptomic and proteomic analyses, Park *et al*. (2021) identified an expanded population of interferon-suppressed plasma B cells in the peripheral blood of fatal SFTS patients. These cells were strongly associated with elevated viral loads and more severe disease, and they harbored actively replicating virus. To validate these findings, the authors performed *in vitro* infection assays using whole blood from healthy donors. B cell subsets—including memory B cells (CD19^+^CD20^+^CD27^+^), plasmablasts (CD19^+^CD20^-^CD38^+^), and plasma cells (CD19^+^CD20^-^CD38^+^CD138^+^)—were susceptible to infection, with plasmablasts demonstrating the highest permissiveness, reinforcing their central role in viral amplification within the circulation [39].

**Fig. 2:**
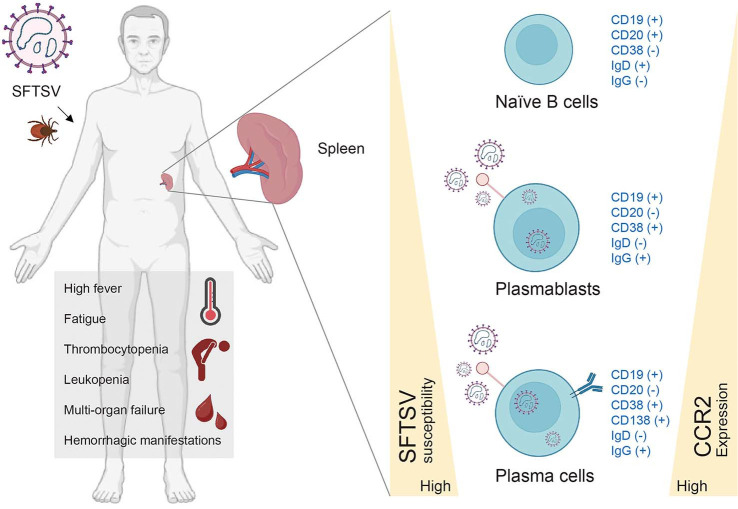
Differentiation of B cell subsets and their susceptibility to SFTSV infection. Naïve B cells (CD19^+^CD20^+^CD38^-^IgD^+^IgG^-^) reside primarily in secondary lymphoid organs such as the spleen and exhibit low susceptibility to SFTSV [38]. Upon activation, B cells undergo class-switch recombination and differentiate into plasmablasts (CD19^+^CD20^-^CD38^+^IgD^-^IgG^+^) and plasma cells (CD19^+^CD20^-^CD38^+^CD138^+^IgD^-^IgG^+^), both of which display increased expression of CCR2. This upregulation of CCR2 correlates with higher SFTSV susceptibility, as CCR2 facilitates viral entry [26, 39]. The diagram illustrates the increasing gradient of CCR2 expression and SFTSV infectivity across B cell maturation stages.

In addition to CCR2 expression on monocytes, Zhang *et al*. (2023) also demonstrated increased expression levels of CCR2 on plasmablasts and plasma cells after their differentiation from B cells. Moreover, H929, a human myeloma plasma cell line with a high level of CCR2 expression, showed significantly higher susceptibility to SFTSV than other B cell lines [26]. These findings indicate that elevated CCR2 expression on monocytes and plasmablasts contributes to poor prognosis and increased mortality in severe SFTS patients. The relationship between B cell differentiation, CCR2 expression, and SFTSV susceptibility is illustrated in Fig. 2.

Collectively, these findings position class-switched B cells—especially plasmablasts and plasma cells—not only as ineffective mediators of protective humoral immunity, but also as permissive hosts that actively amplify viral replication, linking B cell dysfunction to both immunosuppression and disease severity in SFTSV infection.

## T Cell Responses and Dysregulation in SFTSV Infection

T cells play dual roles in SFTSV infection, contributing to antiviral defense but also promoting immunopathology when dysregulated. Clinical and experimental studies have revealed that both CD4^+^ and CD8^+^ T cell populations undergo substantial alterations in number, phenotype, and function over the course of infection.

A pronounced reduction in total T cell counts, particularly CD3^+^ and CD4^+^ subsets, has been consistently observed in patients with severe or fatal outcomes, correlating with disease progression [40]. Further analysis revealed that patients who succumbed to infection exhibited significantly lower absolute counts of Th1, Th2, and regulatory T cells (Tregs) compared to survivors, while Th17 cells remained largely unaffected. Notably, the residual CD4^+^ T cell compartment in these patients was skewed toward Th2 and Th17 lineages, resulting in imbalanced Th2/Th1 and Th17/Treg ratios that were positively associated with adverse clinical outcomes [41]. These findings suggest that both quantitative depletion and qualitative dysregulation of CD4^+^ T cells contribute to disease severity and may offer opportunities for therapeutic intervention.

**Fig. 3:**
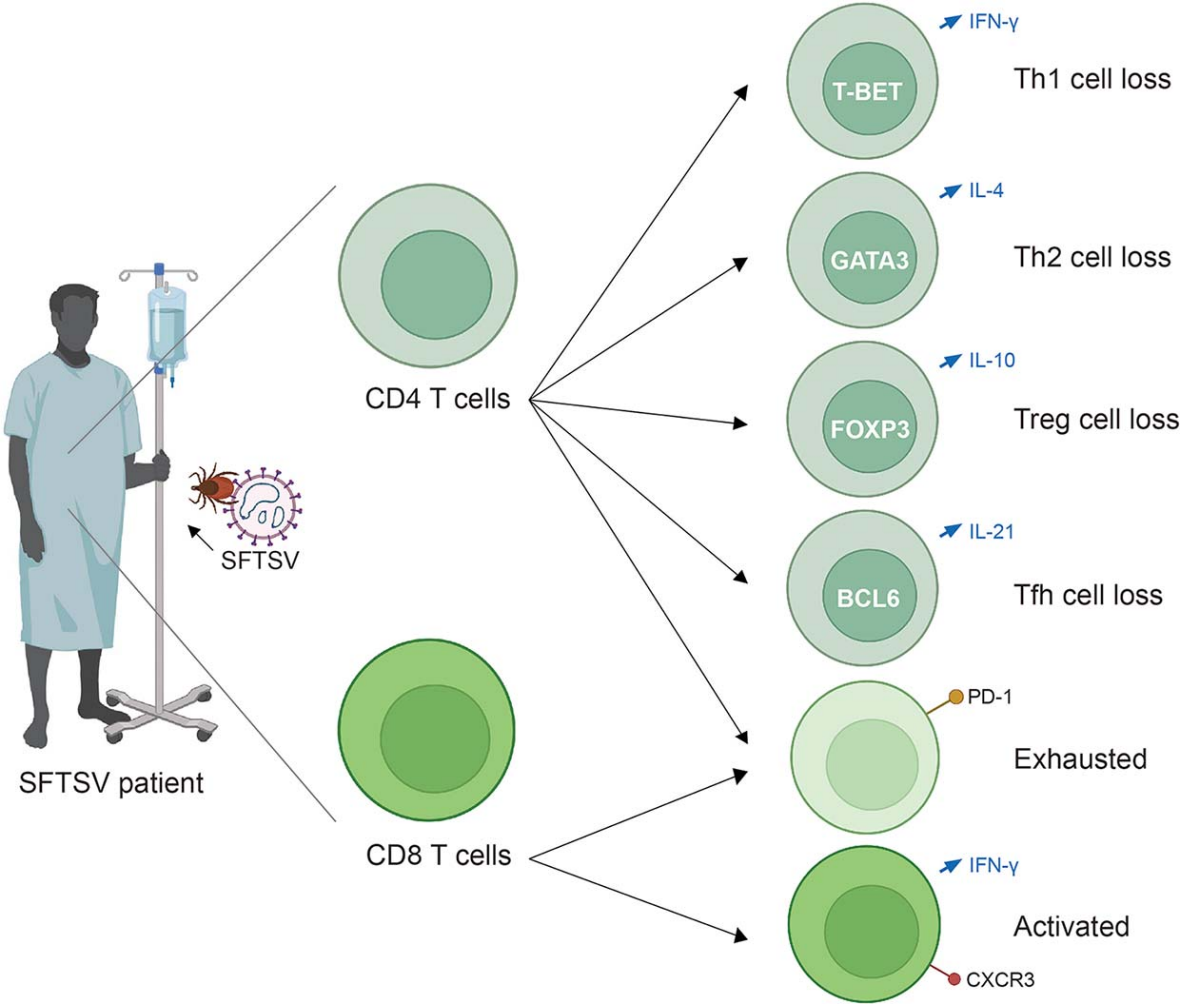
Dysregulated T cell responses in a fatal SFTSV patient. In fatal cases of SFTSV infection, CD4^+^ and CD8^+^ T cells undergo severe quantitative and functional dysregulation [40, 41]. CD4^+^ T cells show marked depletion across multiple subsets, including Th1, Th2, regulatory T (Treg), and follicular helper T (Tfh) cells [23, 41]. CD8^+^ T cells initially respond to infection but subsequently become functionally exhausted, as indicated by PD-1 upregulation [36, 42]. These alterations impair antiviral immunity and contribute to poor clinical outcomes.

In addition to quantitative loss, T cells undergo profound functional exhaustion. Single-cell transcriptomic analyses revealed upregulation of exhaustion markers in both CD4^+^ and CD8^+^ T cells, particularly in fatal cases. Among these, GZMH^+^ and GNLY^+^ effector CD8^+^ T cells emerged as highly exhausted subsets, implicating T cell dysfunction in the failure to control viral replication [36]. T cell exhaustion is typically driven by persistent antigen stimulation and is characterized by sustained expression of inhibitory receptors such as PD-1, LAG-3, and TIM-3, along with reduced cytokine production and cytotoxic activity. In this context, the PD-1/PD-L1 pathway plays a central role in mediating exhaustion by delivering inhibitory signals that suppress effector functions [42]. Supporting this, recent evidence shows that blockade of the PD-1/PD-L1 axis with a nanobody (NbP45) significantly suppressed SFTSV replication in PBMCs, suggesting that releasing PD-1-mediated inhibition can restore T-cell effector activity and improve antiviral responses, likely by reducing apoptosis and enhancing T cell proliferation, underscoring the therapeutic potential of immune checkpoint inhibition [43]. Dysregulated T cell responses during SFTSV infection are illustrated in Fig. 3.

Despite exhaustion, CD8^+^ T cells retain partial antiviral functionality during early infection. SFTS patients demonstrate elevated expression of activation markers (CD25 and CD69), increased IFN-γ and granzyme production, and enhanced proliferation compared to healthy controls—responses which gradually decline during the recovery phase. In parallel, elevated frequencies of CD14^+^CD16^+^ intermediate monocytes and increased expression of CXCL10 and CXCR3 suggest that CXCL10–CXCR3 signaling facilitates CD8^+^ T cell recruitment and activation [44].

T follicular helper (Tfh) cells also appear to play a pivotal role in orchestrating humoral responses. Peripheral Tfh (pTfh) cells—defined as CD3^+^CD4^+^ICOS^+^CXCR5^+^PD-1^+^—expanded markedly during the first week of illness in survivors but failed to expand in fatal cases. Survivors also exhibited a higher frequency of IL-21–producing pTfh cells, with elevated IL-21 mRNA levels in sorted Tfh populations. These data highlight the importance of early and functional T–B cell interactions for effective antibody responses and favorable outcomes [23].

Vaccine studies further emphasize the protective role of T cell immunity. DNA-based vaccination using a single plasmid encoding SFTSV antigens (Gn, Gc, and NP–NS), co-delivered with IL-12 and Flt3L, induced strong CD4^+^ and CD8^+^ T cell responses and provided complete protection in IFNAR-deficient mice—even in the absence of neutralizing antibodies [45]. Similarly, an mRNA vaccine encoding SFTSV Gn induced IFN-γ–producing CD8^+^ T cells, activated Tfh cells, leading to upregulation of CD25^+^ and CD69^+^ activation markers in mice [46]. Likewise, an mRNA vaccine candidate (VER-001) encoding both Gn and Gc elicited robust IFN-γ responses in CD8^+^ T cells, reinforcing the capacity of mRNA platforms to stimulate potent cytotoxic T cell immunity [47].

Taken together, SFTSV infection is associated with both numerical loss and functional impairment of T cells. CD4^+^ T cell depletion and skewed subset differentiation are linked to severe disease, while early CD8^+^ T cell activation and robust Tfh responses are associated with recovery. These insights provide a framework for targeting T cell responses in future therapeutic and vaccine strategies.

## Non-Lymphoid Immune Cells in Host Defense and SFTSV Pathogenesis

Various innate immune cells—such as natural killer (NK) cells, macrophages, dendritic cells (DCs), and even platelets—play essential roles in the host defense and pathogenesis of SFTSV infection.

NK cells are critical innate effectors responsible for early recognition and elimination of virus-infected cells. Clinical studies have demonstrated that reduced frequencies of CD56^dim^CD16^+^ NK cells are associated with increased disease severity in SFTS patients. During the acute phase of infection, these NK cells display elevated expression of Ki-67 and granzyme B, indicative of proliferation and cytotoxic activation, while showing decreased levels of the inhibitory receptor NKG2A, pointing to enhanced effector function [48]. Additionally, another study highlighted the significant reduction of Siglec-9 expression on NK cells in SFTS patients. Siglec-9^+^ NK cells exhibited higher expression of activating receptors and stronger cytolytic activity than their Siglec-9^-^ counterparts. Loss of Siglec-9 expression correlated with impaired NK cell function, suggesting its potential as a biomarker for cytotoxic NK cell subsets in SFTSV infection [49].

Macrophages, derived from activated monocytes, play critical roles in SFTSV pathogenesis by serving as both viral replication sites and mediators of inflammation, immune suppression, and dissemination. Splenic macrophages normally remove aged or damaged platelets from circulation [50, 51]. SFTSV has been shown to bind circulating platelets; this marks them as “altered self ” and promotes their recognition and engulfment by splenic macrophages—a process repeatedly observed in animal models and proposed as a principal mechanism underlying SFTS-associated thrombocytopenia [52, 53]. In murine models, colocalization of virus-bound platelets within splenic macrophages provides *in vivo* evidence for this clearance process, which is further supported by *in vitro* phagocytosis of SFTSV-coated platelets by primary macrophages [52]. In addition, SFTSV infection can trigger platelet activation and apoptosis, exposing clearance signals such as P-selectin and phosphatidylserine that facilitate platelet–macrophage interactions and are associated with platelet activation pathways involving glycoprotein VI (GPVI) [54]. On the macrophage side, the viral NSs protein activates the Nrf2 pathway and enhances expression of the scavenger receptor CD36, thereby enhancing macrophage-mediated clearance of virus-bound platelets [55].

This platelet–macrophage interaction, visualized via confocal microscopy and confirmed *in vitro*, also facilitates viral entry into macrophages, potentially representing an alternative route of infection. While macrophages support early viral replication, the clearance of virus-bound platelets by splenic macrophages may contribute to the splenic viral load reduction at later stages [52]. Postmortem analyses of fatal SFTS cases identified macrophages and plasmablasts as major infected cell types at terminal stages, highlighting their contribution to systemic viral spread [38]. SFTSV also modulates macrophage polarization. During infection, macrophages shift toward an anti-inflammatory M2 phenotype, driven by miR-146a/b upregulation targeting STAT1. Notably, the viral NSs protein enhances miR-146b expression and IL-10 production, promoting M2 differentiation. This phenotypic shift facilitates immune evasion and viral shedding, aggravating disease progression [53].

Dendritic cells (DCs)—particularly myeloid dendritic cells (mDCs)—are central to antigen presentation and the initiation of antiviral adaptive immunity. Both the number and functional capacity of DCs decline significantly during SFTSV infection, correlating with disease severity. SFTSV exploits DC-SIGN via its glycoproteins Gn and Gc to enter target cells, thereby identifying DCs as early sites of viral entry [56, 57]. Clinically, patients with severe or fatal outcomes exhibit a marked reduction in circulating mDCs (CD11c^+^CD123^-^), while survivors show recovery of these cells from the second week post-onset, implicating mDC restoration in favorable outcomes [58]. Functionally, mDCs isolated from deceased patients exhibited reduced expression of Toll-like receptor 3 (TLR3), potentially compromising their ability to recognize viral RNA [58]. Single-cell RNA sequencing further revealed depletion of DCs in SFTS patients compared to healthy controls [36]. Importantly, antigen presentation capacity differs between survivors and fatal cases. Survivors exhibit increased CD86 expression and more frequent CD80^+^CD86^+^ mDCs during weeks 2–3, a pattern absent in fatal cases, indicating deficient activation. *In vitro* experiments further demonstrated that SFTSV-infected mDCs induced lower T cell proliferation and IFN-γ secretion, reflecting functional impairment [23]. Additionally, mDC levels after day 9 were proposed as a prognostic biomarker, showing inverse correlation with IL-6, IL-10, TNF-α, and viral load [59]. DCs from patients also exhibited reduced CD1a and CD83 expression, suggesting defects in phenotypic maturation [60].

**Fig. 4:**
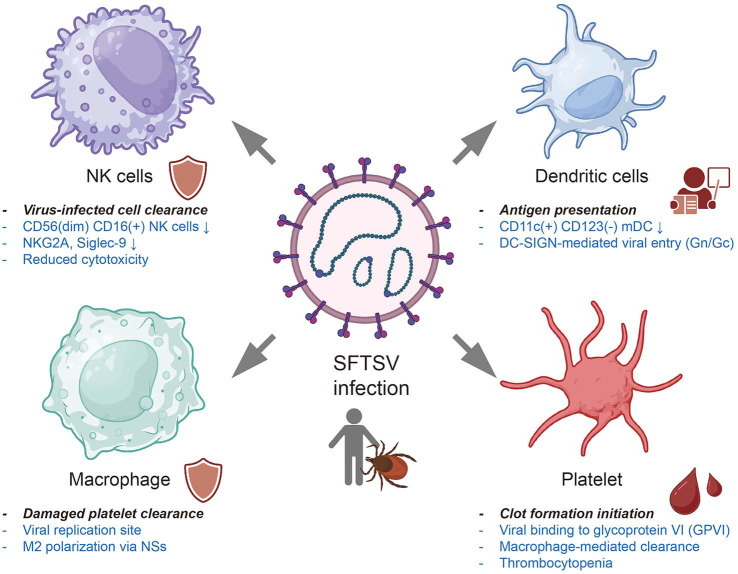
Dysregulated roles of non-lymphoid immune cells during SFTSV infection. NK cells, which are responsible for eliminating virus-infected cells, exhibit reduced frequencies of cytotoxic subsets (CD56^dim^CD16^+^), along with downregulation of NKG2A and Siglec-9, leading to impaired cytotoxic function. DCs, key mediators of antigen presentation and initiators of adaptive immunity, are diminished in both number and function, with SFTSV entry facilitated by DC-SIGN. Macrophages serve as viral replication sites and mediate the clearance of damaged platelets, while skewing toward an M2 phenotype via the viral NSs protein, thereby promoting immune evasion. Platelets initiate clot formation through glycoprotein VI (GPVI) signaling but are also phagocytosed by macrophages, contributing to thrombocytopenia—a hallmark of severe SFTS. Supporting references for this figure are summarized in Table 1.

Thrombocytopenia is a hallmark of SFTSV infection and closely correlates with poor clinical outcomes. While previously attributed mainly to coagulation abnormalities and platelet consumption, recent studies suggest that platelets also play active roles in SFTSV pathogenesis and antiviral defense. A large retrospective analysis of 1,538 SFTS patients revealed that thrombocytopenia correlated with elevated cytokine levels, endothelial injury, and coagulopathy—features strongly linked to poor prognosis. Notably, platelet transfusion failed to improve survival, reduce bleeding risk, or restore platelet counts, highlighting the need for host-directed immunomodulatory strategies over supportive care alone [61]. Fig. 4 illustrates the roles of non-lymphoid immune cells and their dysregulation during SFTSV infection.

Taken together, these findings highlight the complex and context-dependent roles of non-lymphoid immune cells in SFTSV infection. Their contributions span viral clearance, inflammation, immune evasion, and disease progression, offering potential cellular and molecular targets for future therapeutic intervention.

## Interferon Signaling and Host Susceptibility to SFTSV Infection

Type I interferons (IFNs), particularly IFN-α and IFN-β, constitute a central arm of the innate immune response to viral infection [62, 63]. Upon sensing viral components, host pattern recognition receptors (PRRs) activate signaling cascades that lead to the production of type I IFNs, which in turn signal through the IFN-α/β receptor (IFNAR). This receptor engagement activates the JAK-STAT pathway and promotes the transcription of interferon-stimulated genes (ISGs), which collectively restrict viral replication, enhance antigen presentation, and coordinate downstream immune activation [64-66]. Recent ISG screening identified Cyclin D3 (CCND3) as an interferon-induced host restriction factor that suppresses SFTSV replication by binding the viral NP, thereby disrupting ribonucleoprotein complex formation. The viral NSs protein counteracts this effect by downregulating CCND3 and promoting its degradation, underscoring a dynamic balance between interferon-mediated restriction and viral counteraction [67]. However, SFTSV has evolved multiple mechanisms to evade and suppress these antiviral defenses.

Initial studies revealed that SFTSV infection induces IFN and ISG expression, but this response is short-lived and ultimately fails to control viral replication. One investigation showed that key upstream molecules, such as mitochondrial antiviral signaling protein (MAVS), are downregulated following infection. Moreover, activation of IRF and NF-κB pathways appears dampened, suggesting that early innate immune sensing is actively inhibited [68]. The SFTSV nonstructural protein NSs, encoded by the S segment, functions as a key antagonist of type I IFN signaling [69]. NSs interacts with TBK1—a kinase upstream of IRF3—and sequesters it into viral inclusion bodies (IBs), thereby preventing IRF3 phosphorylation and its nuclear translocation, leading to suppressed IFN-β production [70]. Structural studies further revealed that deletion of the N-terminal 25 amino acids in NSs abrogated its IFN-suppressive function, indicating a critical functional domain.

NSs also binds RIG-I and TBK1, further impairing early viral recognition and IRF3 activation by sequestering these molecules into IBs, thereby impairing the RIG-I–TBK1–IRF3 signaling axis. This sequestration not only disrupts early viral recognition but also blocks downstream IRF3 activation and nuclear translocation, contributing to the overall shutdown of IFN-I induction [27].

Beyond IRF3 pathway blockade, NSs also targets downstream components of the JAK-STAT cascade. NSs binds directly to STAT1 and STAT2, blocking their phosphorylation and nuclear translocation, thereby silencing downstream ISG expression. One study demonstrated that NSs sequesters STAT1/2 into cytoplasmic IBs and inhibits ISG expression even in the presence of exogenous IFN stimulation [71, 72]. Co-immunoprecipitation and confocal microscopy confirmed that the DNA-binding domain of STAT2 facilitates its interaction with NSs, resulting in retention within cytoplasmic compartments and failure of antiviral gene activation.

Importantly, the antagonistic role of NSs extends beyond type I IFNs to type II IFN (IFN-γ) signaling as well. IFN-γ is produced predominantly by activated T cells and NK cells and plays a critical role in macrophage activation and intracellular pathogen control [73]. High serum levels of IFN-γ were observed in SFTS patients and shown to suppress SFTSV replication in *in vitro* models. However, the efficacy of IFN-γ was compromised after infection was established. Mechanistically, NSs directly binds to STAT1—the key transcription factor in the IFN-γ pathway—and sequesters it into IBs. Additionally, NSs downregulates STAT1 protein levels, thereby inhibiting IFN-γ-mediated signaling and antiviral activity [74]. In murine models, pre-infection IFN-γ treatment markedly improved survival, while post-infection administration failed to confer protection, emphasizing the importance of early interferon responses and the timing of intervention.

These robust immune evasion mechanisms likely account for the limited efficacy of IFN-based therapies in clinical contexts. A retrospective study evaluating IFN-α therapy in SFTS patients found no improvement in survival or clinical outcomes [36], reinforcing the notion that SFTSV’s multifaceted suppression of IFN signaling undermines the efficacy of exogenous IFN administration.

To validate these mechanisms *in vivo*, researchers have employed mouse models with impaired IFN responses. In a seminal study, IFNAR1 knockout (IFNAR^-^/^-^) mice were highly susceptible to SFTSV infection, succumbing within 3–4 days post-inoculation. Viral antigen was widely detected across multiple organs, including spleen, lymph nodes, liver, and brain, indicating systemic viral dissemination in the absence of IFNAR signaling [30, 75]. Interestingly, wild-type (WT) mice remained asymptomatic, underscoring the protective role of IFN-I.

Subsequent studies introduced a transient IFNAR1 blockade model using neutralizing antibodies (IFNAR Ab). Unlike IFNAR^-^/^-^ mice, which succumb rapidly to infection, IFNAR Ab-treated mice exhibited intermediate susceptibility. High-dose viral challenge resulted in mortality, whereas lower doses permitted survival and recovery [31, 76]. This model more accurately mimics the partial IFN suppression observed in human SFTS and offers a tractable system for evaluating pathogenesis and interventions.

Taken together, these findings highlight the central role of IFN signaling—particularly the STAT1/2 axis and TBK1–IRF3 pathway—in restricting SFTSV infection. The virus uses its NSs protein to hijack and immobilize host signaling components within cytoplasmic inclusion bodies, thereby disabling the antiviral machinery. In parallel, mouse model studies reinforce the importance of IFNAR signaling in controlling viral spread and pathogenesis. Elucidating these interactions lays the groundwork for rational design of therapeutics that restore IFN signaling or bypass viral immune evasion mechanisms.

Collectively, findings across immune cell subsets indicate that SFTSV pathogenesis is driven not by isolated molecular events, but by coordinated reprogramming of virus-permissive immune cells that serve as viral reservoirs and amplifiers of immunopathology. At the molecular level, these cellular abnormalities converge on interferon signaling pathways antagonized by the viral NSs protein, providing a unifying explanation for the limited efficacy of immune-modulatory therapies. Rather than cataloging individual molecular pathways, this integrative framework links immune cell-specific viral targeting with immune evasion and therapeutic failure during SFTSV infection. To provide an integrated overview of immune cell-oriented responses and interferon signaling discussed throughout this review, the key findings are summarized in Table 1.

## Conclusion and Therapeutic Implications

SFTS is a complex viral disease where immune cell infection, dysfunction, and cytokine dysregulation converge to drive severe pathology and high mortality. This review outlines how SFTSV disrupts antiviral immunity by targeting key immune cell populations—including monocytes, B cells, T cells, NK cells, and dendritic cells—creating a paradoxical state of inflammation and immunosuppression. In particular, monocytes and plasmablasts emerge as key viral reservoirs, while CD4^+^ and CD8^+^ T cells undergo numerical depletion, functional exhaustion, and skewed subset polarization in fatal cases. Moreover, the depletion and dysfunction of mDCs contribute to impaired antigen presentation, further dampening adaptive responses.

A central finding across numerous studies is the potent immune evasion strategy employed by the SFTSV nonstructural protein (NSs). This viral protein disrupts host antiviral defenses by sequestering critical signaling molecules—including TBK1, IRF3, STAT1, and STAT2—into viral inclusion bodies and thereby disrupting host antiviral defenses. These interactions prevent the activation of type I and II interferon pathways, thus subverting both innate and adaptive immunity. Animal studies using IFNAR^-^/^-^ mice or antibody-mediated IFNAR blockade further confirm the essential role of interferon responses in controlling viral dissemination and host survival.

Recent advances have uncovered several promising immunotherapeutic approaches against SFTSV. Notably, an alpaca-derived anti-PD-1 nanobody (NbP45) exhibited strong antiviral activity both *in vitro* and in SFTSV-infected humanized mice, likely due to enhanced tissue penetration and reduced viral escape, thereby offering superior protection over conventional IgG antibodies [43]. Similarly, IFN-γ pretreatment, but not post-infection administration, protected mice from lethal SFTSV challenge, highlighting the importance of early intervention and the virus’s ability to evade IFN-γ–mediated immunity post-infection. Blockade of the CCR2–CCL2 axis—which regulates monocyte and B cell recruitment and facilitates viral entry through direct interaction with SFTSV glycoprotein Gn—also reduced viral replication and disease severity in mice, positioning CCR2 as both a viral entry receptor and a promising therapeutic target.

In parallel, vaccine-based strategies have shown substantial potential. A recent study demonstrated that a lipid nanoparticle-encapsulated mRNA vaccine encoding SFTSV Gn induced robust neutralizing antibody and T cell responses in mice, including IFN-γ–producing CD8^+^ T cells and activated Tfh cells, conferring complete protection against lethal SFTSV challenge [46]. Building on this, another mRNA vaccine candidate (VER-001), which encodes both Gn and Gc proteins, similarly promoted strong cellular immunity—marked by elevated IFN-γ–producing CD8^+^ T cells—underscoring the versatility and immunogenic strength of mRNA-based platforms against SFTSV [47]. Likewise, a DNA-based vaccine encoding SFTSV antigens (Gn, Gc, and NP-NS), co-administered with IL-12 and Flt3L, achieved complete protection in IFNAR-deficient mice. Notably, protection was mediated by strong CD4^+^ and CD8^+^ T cell responses, even in the absence of neutralizing antibodies—highlighting the importance of cell-mediated immunity.

Despite recent progress in SFTSV immunopathogenesis, how virus-permissive immune cells are reprogrammed and sustained *in vivo*—and how this drives disease progression and therapeutic failure—remains incompletely understood. Future research should focus on three priorities: (1) disrupting NSs–host protein interactions to restore interferon signaling; (2) reversing T cell exhaustion and enhancing adaptive immunity—including antigen presentation and antibody production; and (3) developing vaccines or monoclonal antibodies targeting the Gn and Gc envelope glycoproteins to block viral entry. Importantly, murine models that recapitulate partial interferon suppression—such as IFNAR antibody-treated mice and mouse-adapted SFTSV infection models—provide valuable platforms for preclinical testing of these interventions.

In summary, SFTSV employs a multifaceted strategy of immune modulation involving cellular tropism, immune suppression, and signaling blockade. A deeper understanding of these mechanisms not only clarifies disease pathogenesis but also informs the rational design of targeted therapies. Ultimately, integrating insights from viral biology with host-targeted immunomodulatory strategies holds the greatest promise for combating this emerging infectious threat.

## Figures and Tables

**Table 1:** Overview of immune cell-associated responses and interferon signaling in SFTSV infection.